# Multi-omics analysis of *HLTF* context-dependent regulation across TCGA cohorts revealed its potential as new biomarker in tumour

**DOI:** 10.1007/s00335-026-10257-w

**Published:** 2026-07-24

**Authors:** Alessia Canevotti, Marija Tursunović, Wendalina Tigani, Graziano Pesole, Matteo Chiara, Matteo De March

**Affiliations:** 1 Dipartimento di Bioscienze, Università degli Studi di Milano Statale, via Celoria 26, 20133 Milano, Italy; 2 Istituto di Biomembrane, Bioenergetica e Biotecnologie Molecolari, Consiglio Nazionale delle Ricerche, via Amendola 122o, 70126 Bari, Italy; 3 Innovative Centre of the Faculty of Chemistry, University of Belgrade, Studentski trg 16, 11158 Belgrade, Serbia; 4 Department of Life Sciences, University of Trieste, via Licio Giorgieri 5, 34100 Trieste, Italy; 5 Dipartimento di Bioscienze, Biotecnologie e Ambiente, Università di Bari Aldo Moro, via Orabona, 70125 Bari, Italy; 6 Laboratory for Environmental and Life Sciences, University of Nova Gorica, Vipaska 13, SI-5000 Nova Gorica, Slovenia

## Abstract

The SNF2-family Helicase-Like Transcription Factor (HLTF) has been implicated in tumorigenesis, with evidence suggesting both tumour-suppressive and oncogenic functions. To clarify the nature of this increasingly emerging double function, we analysed the full *HLTF’s *alterome across TCGA cohorts combining first gene expression with genomic and epigenomic layers of de-regulation, and investigating further their determinants through the integration of DNA methylation profiles - both at promoter-proximal and distal regions, the copy-number alterations, and all somatic mutations. This multi-layered analysis highlighted specific context-dependent* HLTF’s* regulatory configurations across human cancers, even modulated by either dosage effects or loss-of-function events. The clinical relevance of *HLTF’s* alterations, identified through tumour-specific associations with patient outcomes, supports its potential as new biomarker in cancer overall.

**Supplementary Information:**

The online version contains supplementary material available at 10.1007/s00335-026-10257-w.

## Background

Cancer is a multifactorial disease arising from the interplay of genetic alterations, epigenetic changes, and environmental factors, which together disrupt cellular homeostasis and drive the formation and proliferation of malignant cells (Zhang et al. 2024). Cancer development and progression are shaped by genetic alterations and epigenetic dysregulation affecting key regulators of genome stability, transcription, and DNA repair (Kontomanolis et al. 2020). Among these, the Helicase-Like Transcription Factor (*HLTF*), a member of the SNF2-family of chromatin remodellers with key functions in gene transcription and genome stability, has recently emerged as a candidate player in tumorigenesis (Waheed et al. 2024).

A recurrent theme in the literature is the context-dependent behaviour of *HLTF* and its possible implications in cancer. Decreased *HLTF* activity was associated with an increase in stalled replication forks, sustained DNA damage, and genome instability, compatible with a role in the maintenance of genome stability/integrity (Takaoka et al. 2019; Bai et al. 2020). Down-regulation of *HLTF* through promoter hypermethylation was reported to correlate with poor patient prognosis in colorectal cancer (Sandhu et al. 2012; Moinova et al. 2002), cervical adenocarcinoma (Kang et al. 2006) and gastric carcinoma (Hamai et al. 2003), thus supporting an oncosuppressor role.

Nevertheless, a recent study indicates that *HLTF* can transcriptionally activate the serine protease inhibitor clade E member 1 (SERPINE1) in cervical cancer cells (CC), thereby promoting malignant transformation from precancerous lesions to CC (Wang et al. 2025). Additionally, in hepatocellular carcinoma (HCC), *HLTF* enhances the stability of the serine/arginine-rich splicing factor 1 (SRSF1), resulting in the activation of the ERK/MAPK signalling pathway, which, in turn, promotes to cell proliferation and metastasis (Xu et al. 2023).

Therefore, based on this evidence *HLTF* may function as a double agent in cancer, similarly to other functionally analogous proteins (He et al. 2018; Li et al. 2023). The recently reported role of *HTLF* in transcriptional modulation, either by direct binding at gene promoter, as in the case of β-globin (Mahajan and Weissman 2002), or by interacting with other transcription factors such as Sp1/Sp2 (Ding et al. 1999), is not incompatible with this model. Moreover, *HLTF* implication in the regulation of the circadian prolactin gene expression, and the melanosome transmembrane protein (Guillaumond et al. 2011; Visser et al. 2012) further suggests its propension to a broader target repertoire consistent with functional multivalency.

Despite these hypotheses, to our knowledge no systematic assessment has yet evaluated the nature and impact of *HLTF* alterations across cancer types. This gap prevents any definitive conclusion about its prognostic value.

Large-scale cancer genomics resources such as The Cancer Genome Atlas (TCGA) (Yao et al. 2024) provide an opportunity to systematically characterize *HLTF* across diverse tumour types under a unified analytical framework.

Here, we present an integrative analysis of *HLTF* alterations across TCGA cohorts, combining gene expression with DNA methylation profiles at promoter-proximal and distal regions, copy-number alterations, and somatic mutations. We then evaluate the clinical relevance of *HLTF* alterations through tumour-specific survival analyses. By integrating genomic and epigenomic evidence, we provide a comprehensive view of *HLTF* regulation across 33 distinct cancer types and support a model in which distinct regulatory modes contribute to its context-dependent behaviour which was only partly described in previous studies (Arcolia et al. 2014; Dhont et al. 2018).

## Methods

### Data collection

We analysed cohorts from the TCGA Pan Cancer Atlas using uniformly processed data by the TCGA consortium (Cerami et al. 2012; Goldman et al. 2020).

Specifically, the gene expression values Log_2_(TPM) (TPM: Transcript Per Million) were obtained via UCSC Xena (Goldman et al. 2020); a pseudocount of 0.0001 was added to TPM to prevent undefined values. DNA methylation β-values for the probes cg26151310, cg04836786, cg02059813 and cg21089667 (cBioPortal (Cerami et al. 2012)) derived from the Illumina HumanMethylation450 array were obtained from UCSC Xena (Goldman et al. 2020). The regulatory elements annotation was derived from Ensembl regulatory build (Zerbino et al. 2015). Copy Number Alterations data (CNAs: HomDel, HetLoss, Diploid, Gain, Amp) according to *GISTIC2* (Mermel et al. 2011) were retrieved from UCSC Xena. The full list of somatic variants (SNVs and indels) was obtained from cBioPortal (Cerami et al. 2012). We retrieved the Overall Survival (OS) data via UCSC Xena (Goldman et al. 2020) and GEPIA2 (Tang et al. 2017).

The total number of samples available across all cancer types, together with each omics-assay considered in this study are reported in the Supplementary Table S1.

### mRNA expression

We analysed only tumour types for which at least 9 matched tumour-normal sample pairs were available, for a total of 653 samples with paired data across 15 distinct tumour types (Supplementary Table S2). A Kolmogorov–Smirnov test (Massey 1951) was applied to assess the statistical significance of changes in *HLTF* expression differences between tumour and normal samples. Changes in expression were visualized in the form of the log fold change (FC) of expression values: Log_2_(TPM_tumour_/TPM_normal_).

### Identification of cancer types with altered HLTF methylation

All β-values reporting the distribution of methylation status probed both at promoter-proximal (cg26151310, cg04836786) and promoter-distal (cg02059813, cg21089667) regions [21] were inspected and arbitrarily discretized. For proximal probes, β-values < 0.1 were labelled hypomethylated and β-values > 0.1 non-hypomethylated. For distal probes, we categorized β-values > 0.9 as hypermethylated and β-values < 0.9 as non-hypermethylated.

Samples carrying CNAs at the *HLTF* locus were excluded from methylation analyses to avoid bias in β-values calculation (Aure et al. 2013; Sun et al. 2018; Shi et al. 2020).

To identify cancer types showing an increased proportion of samples with dysregulated *HLTF* methylation, for each tumour type, we calculated the fraction of samples falling into each methylation class (hypomethylated, non-hypomethylated, hypermethylated, and non-hypermethylated). Each tumour type was then contrasted against a background cohort defined as the pooled set of samples across all the other tumour types. For the promoter-distal probes we compared the number of hypermethylated *versus* non-hypermethylated samples; whereas hypomethylated *versus* non-hypomethylated were compared for promoter-proximal probes. A Fisher’s exact test (one-sided, “greater”), as implemented in the *fisher.test* (Core Team 2020), was used to detect statistically significant variations (Fisher 1922). In the visual representation of the data, p-values below 1e^− 16^ were capped at this threshold before applying a logarithmic transformation.

The statistical significance of each test was attributed with *p* ≤ 0.05, adjusted using the Benjamini Hochberg’s procedure (FDR value) to control the false discovery rate. The heatmaps were generated using the pheatmap package (Core Team 2020). All data and results can be inspected in the Supplementary Tables S3 and S4.

### Correlation of expression with methylation

We used Pearson’s correlation coefficient to evaluate the linear correlation between β-values and *HLTF* expression (Log_2_(TPM + 0.001)) (Massey 1951), fit the data with a linear model (*lm* function (Core Team 2020)), and compute the statistical significance (*cor.test* (Core Team 2020)) (Supplementary Table S5).

### Copy number alterations

We defined five distinct CNA categories, based on GISTIC2 categorization (Cerami et al. 2012; Mermel et al. 2011), namely (i) HomDel: deletion of both copies of the gene, ii) HetLoss: deletion of one copy of the gene, iii) Diploid: no CNA, iv) Gain: acquisition of a single extra copy of the gene, and v) Amp: acquisition of multiple extra copies of the gene (Supplementary Table S6).

To identify those cancer types containing an increased proportion of CNAs, we built a 2 × 2 contingency table by recording the total number of samples with these alterations and the total number of diploid samples (Supplementary Table S7). Each tumour type was then evaluated against the aggregated counts observed across all other tumour types. Fisher’s exact test (Fisher 1922) was applied to identify those tumours with a statistically significant enrichment of each CNA. Because we observed recurrent CNAs at the *HLTF* locus, we assessed whether these alterations could involve upstream and downstream genes, including: *HPS3*, *CP*, and *TM4SF18* (downstream), and *GYG1*, *CPA3*, and *CPB1* (upstream). Upstream and downstream genes were established according to the RefSeq annotation 10.4 of the GRChg19 human genome (Perez et al. 2025).

### Gene dosage analysis

To evaluate the effect of CNAs on *HLTF* expression within each tumour type we grouped samples by each CNA category and compared their observed expression values with the mean *HLTF* expression observed for diploid samples. A total of 1534 samples, with no retrievable expression data, were excluded. The expression CNA_samples_/CNA_diploid−average_ was calculated as Log_2_(FC). The differences between each group (i.e., Diploid *versus* HetLoss, Diploid *versus* Gain) were evaluated using the two-sample Kolmogorov–Smirnov test (Massey 1951). Consistent with our mRNA expression analysis, only tumours for which a sufficient number of matched normal paired samples (*n* = 9 or greater) were included in this analysis. All details are reported in Supplementary Tables S8 and S9.

### SNVs and short indels

We grouped somatic mutations in two categories based on their predicted functional effect, *deleterious and low-impact*. The *deleterious* included frameshift, nonsense, splice-site mutations, as well as non-synonymous mutations predicted as deleterious by at least two out four methods for the assessment of the effect of non-synonymous substitutions, namely Mutation Assessor (MA) (Choudhury et al. 2021), Sorting Intolerant From Tolerant (SIFT) (Sim et al. 2012), Polymorphism Phenotyping v2 (PolyPhen-2) (Ng 2003; Reva et al. 2011; Adzhubei et al. 2013), and AlphaMissense (Cheng et al. 2023). The *low-impact* included all the other somatic mutations. Tumour types with an increased proportion of *deleterious HLTF* mutations, were identified by applying a Fisher’s exact test to a 2× 2 contingency table comparing their distribution in a specific tumour type, against the aggregated counts of all other tumour types. All these data are summarized in the Supplementary Tables S10 and S11.

### Survival analysis

We used Kaplan–Meier plot (*survfit* function (Core Team 2020)) to evaluate the prognostic effects (overall survival - OS) of molecular signatures derived from the analysis of the different omics layers. Each plot was generated using the *survminer: ggsurvplot* function (Core Team 2020). Statistical significance was attributed using the log-rank test (*survdiff* function (Core Team 2020)). P-values were corrected for multiple testing using the Benjamini-Hochberg for the control of the False Discovery Rate (FDR); a p-value threshold of 0.05 was used for statistical significance. The association of *HLTF* expression levels with OS within the same cancer type were evaluated through *GEPIA2* web interface (*gepia2.cancer-pku.cn)* with default parameters (Tang et al. 2017).

#### Data interpolation and genome assembly

Data were integrated by considering gene level annotation/values when available. Proximal *HLTF* regulatory elements and upstream and downstream genes were visually inspected by considering the hg19 reference assembly of the human genome through the UCSC genome browser.

## Results

To obtain a comprehensive view of *HLTF* dysregulation across cancer types, we performed an integrative analysis of TCGA cohorts combining transcriptomic, epigenomic and genomic layers. We first quantified *HLTF* expression changes across tumour types and observed heterogeneous, cancer-type-specific patterns of deregulation. We then investigated potential determinants of these differences by integrating DNA methylation profiles at promoter-proximal and distal regulatory regions, copy-number alterations, and somatic mutation patterns. Finally, we assessed the prognostic relevance of *HLTF* alterations using tumour-specific survival analyses. We identified distinct regulatory configurations associated with *HLTF* activity across cancers and highlighted tumour contexts in which *HLTF* alterations show the strongest outcome associations.

### *HLTF* is differentially modulated in cancer

**Fig. 1:**
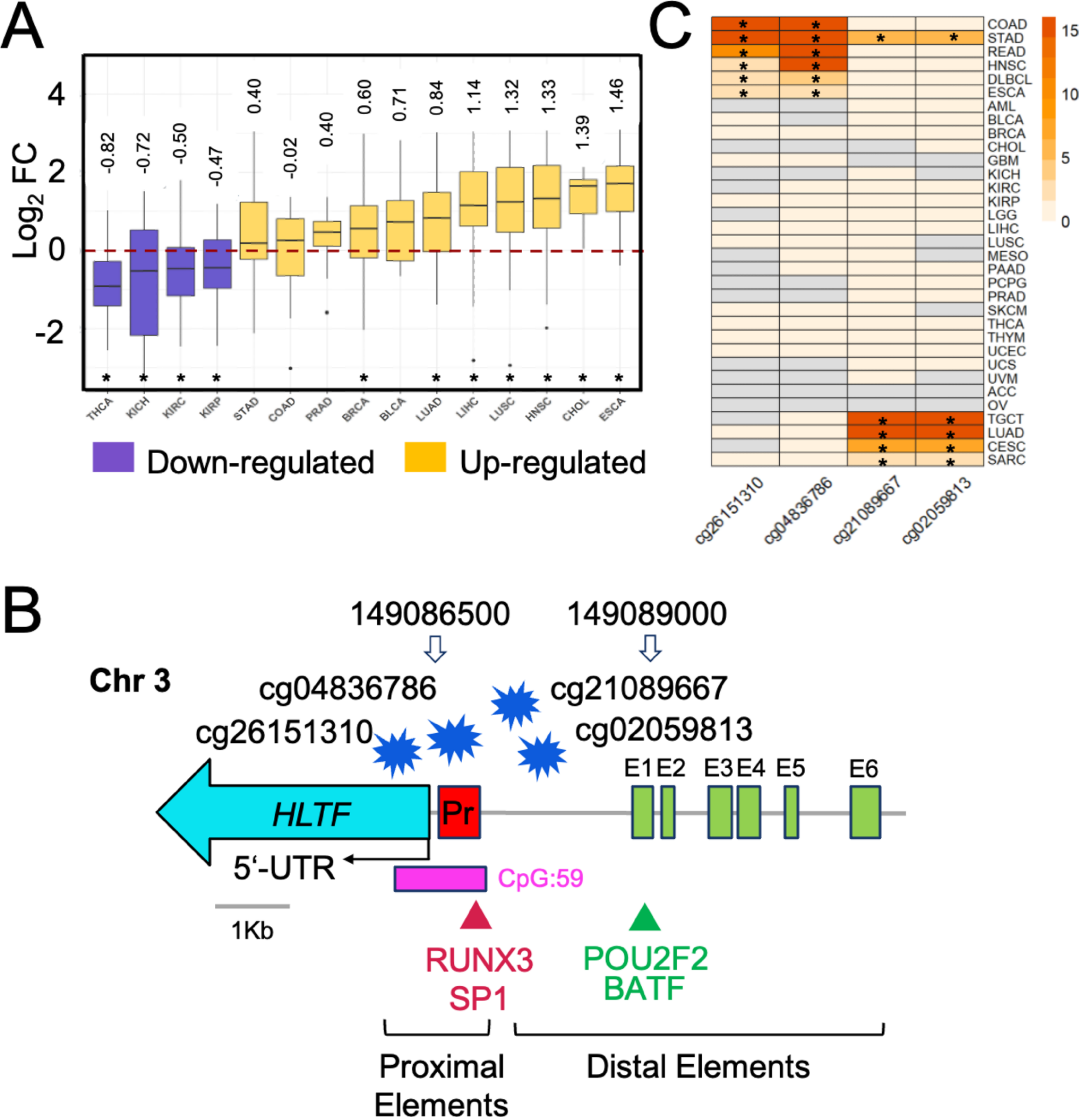
**A** Change of *HLTF* mRNA expression in the 15 cancer types with matched normal tissues. The down- (purple) and up- (yellow) regulation are represented with boxplots as the median Log_2_(FC) (numbers above). Statistically significant differences between tumour and matched normal tissues are marked as * (Supplementary Table S2). **B** Schematic representation of *HLTF* regulatory regions: the gene itself (cyan arrow) and its 5’-UTR, the promoter (Pr, red), the large CpG island (magenta) recognized by RUNX3 and SP1 transcription factors (TFs, triangle dark red), the distal enhancers (E1-E6, green) and their POU2F2 and BATF TFs binding site (triangle dark green). The four methylation probes are represented as blue callouts. Two arrows indicate the corresponding region of the chromosome in bp. **C.** Heatmap of *HLTF *methylation. The p-values for enrichment of either non-hypomethylated samples at the proximal probes (cg26151310, cg04836786) or non-hypermethylated samples at the distal probes (cg21089667, cg02059813) across all cancer types are capped at 1×10^− 16^ to facilitate visualization. The colour scale indicates statistical significance computed as -log10(p-value). Darker colours represent lower p-values

*HLTF* expression is significantly de-regulated in 12 out of the 15 cancer types compared to matched normal tissues, indicating a systematic dysregulation of this gene in cancer (Fig. 1A; Supplementary Table S2). Notably, the direction of deregulation is heterogeneous, with subsets of cancers showing decreased *HLTF* expression and others showing increased expression.

*HLTF* is significantly downregulated in thyroid carcinoma (THCA, log_2_FC −0.82), and in kidney cancers including chromophobe (KICH, log_2_FC −0.72), clear-cell (KIRC, log_2_FC −0.50), and papillary-renal carcinoma (KIRP, log_2_FC −0.47), consistent with previous findings (Arcolia et al. 2014; Debauve et al. 2008). In contrast, *HLTF* is significantly up-regulated in liver hepatocellular carcinoma (LIHC, log_2_FC 1.14) (Fig. 1A; Supplementary Table S2), and in several additional tumour types, including: lung squamous-cell carcinoma (LUSC, log_2_FC 1.32), head and neck squamous-cell carcinoma (HNSC, log_2_FC 1.33) (Capouillez et al. 2009), cholangiocarcinoma (CHOL, log_2_FC 1.39), prostate adenocarcinoma (PRAD, log_2_FC 0.40), breast invasive carcinoma (BRCA, log_2_FC 0.60), and lung adenocarcinoma (LUAD, log_2_FC 0.84) (Fig. 1A; Supplementary Table S2).

We next assessed the prognostic relevance of *HLTF* expression using tumour-specific survival analyses. In KIRC, reduced *HLTF* expression was associated with worse prognosis (Supplementary Figure S1A), whereas in KICH lower expression correlated with improved survival (Supplementary Figure S1B). In LIHC, decreased *HLTF* expression was associated with better prognosis (Supplementary Figure S1C), while no significant association was detected in other tumour types.

### Epigenetic regulation of *HLTF*: promoter methylation and distal regulatory regions

DNA methylation was assessed using TCGA Illumina HumanMethylation450 (450 K) array profiles and reported as β-values (Materials and Methods). We focused on four *HLTF*-associated CpG probes located in promoter-proximal and promoter-distal regions (Fig. 1B). The two *HLTF* promoter-proximal probes, cg26151310 and cg04836786, map to a large CpG island spanning from 235 bp upstream to the transcription start site (TSS) to ~ 200 bp into the first intron, a region reported to be regulated by a limited set of well-known transcription factors (TFs) (Ding et al. 1999; Cheng et al. 2016). In contrast, the promoter-distal probes, cg21089667 and cg02059813, map ~ 1000 bp upstream of the TSS and ~ 1500 to 10,000 bp downstream to 6 annotated enhancers (E1-E6 in Fig. 1B). These enhancers are predicted to contain binding sites for POU2F2 (Luo et al. 2021) and BATF (Lin et al. 2021) (Fig. 1B), although direct regulatory interactions with *HLTF* have not been experimentally established.

Across the full dataset, *HLTF* mRNA levels show a strong inverse correlation with the methylation values recorded by the promoter-proximal probes a (p-value < 2 × 10^− 16^), consistent with transcriptional repression. Promoter-distal probes display a weaker and probe specific pattern: cg02059813 shows a limited yet statistically significant anti-correlation with *HLTF* expression (p-value < 0.02), while for cg21089667 no significant correlation can be detected (Supplementary Table S5; Supplementary Figure S2).

These differences are consistent with distinct methylation regimes at proximal versus distal sites. Promoter-proximal probes are largely unmethylated with ~ 96% of samples having β-values between 0 and 0.1, whereas promoter-distal probes are highly methylated with β-values > 0.9 in ~ 92% of the samples (Supplementary Tables S3; S4 and Supplementary Figure S2). These extreme methylation levels are typically associated with transcriptional silencing, which may partially explain the observed lack of anti-correlation with *HLTF* expression (Supplementary Table S5; Supplementary Figure S2).

We next evaluated tumour-type-specific methylation patterns. A significant excess of tumour samples with elevated promoter-proximal methylation was observed in COAD, stomach adenocarcinoma (STAD), rectum adenocarcinoma (READ), head and neck squamous-cell carcinoma (HNSC), esophageal carcinoma (ESCA), and lymphoid neoplasm diffuse large B-cell lymphoma (DLBCL) (Fig. 1C; Supplementary Table S12). In these cohorts increased promoter methylation was consistently associated with reduced *HLTF* mRNA levels (Supplementary Figures from S3A to F), consistent with previous describing reports of epigenetic dysregulation in colorectal and gastric cancers (Moinova et al. 2002; Hamai et al. 2003; Liu et al. 2018). However, in our analysis we did not detect a robust association with overall survival, likely due to limited sample sizes.

A statistically significant excess of samples with decreased methylation at the promoter-distal probes was observed in testicular germ cell tumour (TGCT), cervical squamous cell carcinoma (CESC), LUAD, STAD and sarcoma (SARC) (Fig. 1C; Supplementary Table S13), but with heterogeneous transcriptional consequences: specifically, *HLTF* is significantly up-regulated in LUAD and CESC, but not in TGCT and STAD, and down-regulated in SARC (Supplementary Figures from S3G to M).

In these tumour types, changes in methylation levels, and associated alterations in *HLTF* mRNA expression (if any), did not show any correlation with overall survival (Supplementary Tables S12, S13). Taken together, these results indicate that promoter-proximal methylation is robustly linked to *HLTF* repression across cancers, whereas methylation at distal regulatory sites likely reflects context-dependent regulatory activity rather than a uniform silencing mechanism.

### Copy-number alterations at the *HLTF* locus show asymmetric dosage effects

**Fig. 2:**
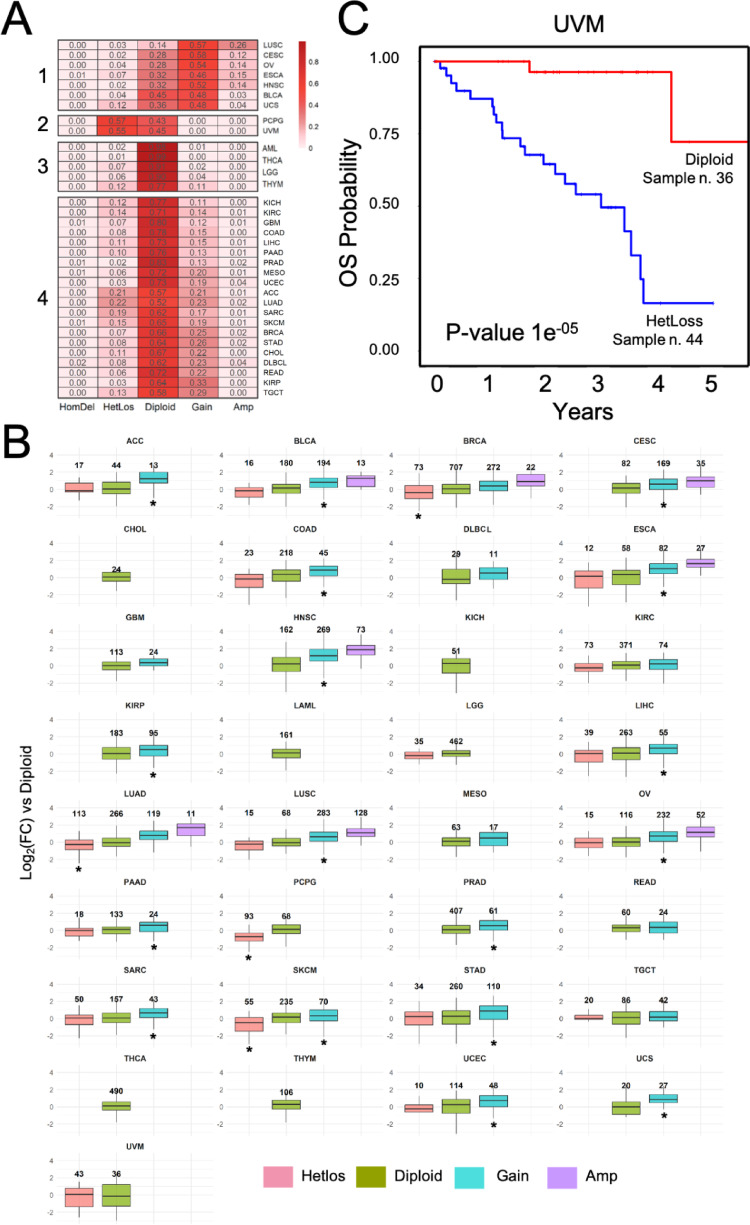
**A** Heatmap of *HLTF* CNAs across 33 cancer types. The colour scale (right) refers to the relative enrichment of each CNA category with respect to the Diploid condition within a given tumour type (Material and Method). The heatmap is divided in four main patterns containing those cancers with preferential *HLTF* amplification (1), preferential heterozygous loss (2), comparable proportions of both cases (3), and low overall CNA frequency (4). **B** Change of *HLTF* mRNA expression represented as Log_2_(FC) relative to diploid samples for each CNA category and across all 33 cancer types. *indicates either the statistically significant decrease of HLTF expression following HetLoss event (Supplementary Table S8) or the statistically significant increase of HLTF expression following Gain event (Supplementary Table S9). **C** Kaplan–Meier plot of Overall Survival (OS) extracted considering both *HLTF* Diploid (blue) and HetLoss (red) conditions in UVM; sample numbers and the overall p-value are reported

Across the pan‑cancer cohorts, CNAs at the *HLTF* locus are common with only ~ 60% of the samples retaining a diploid status (Fig. 2A). Although significant enrichment of *HLTF* CNAs is detected only in a subset of tumour types, CNA patterns across the 33 analysed cancers can be grouped into four recurrent categories (Fig. 2A; Supplementary Table S7).

Category (1) includes the preferential *HLTF* amplification or gain (seven tumour types); category (2) includes the preferential heterozygous *HLTF* loss (two tumour types); category (3) includes the balanced heterozygous gain and loss (20 tumour types); category (4) includes the low CNA frequency (four tumour types).

We next assessed the relationship between *HLTF* copy number and transcript abundance. Overall *HLTF* CNAs have a detectable impact on *HLTF* mRNA expression (Fig. 2B; Supplementary Table S14): whereas loss of a single copy results in a reduced mRNA level relative to the diploid state, the gain of *HLTF* results in a proportional (to the number of copies) *HLTF* expression. This pattern strongly supports a dosage-dependent regulation of *HLTF*, suggesting a dosage effect of CNAs on *HLTF* transcript levels (Hose et al. 2015; Bravo-Estupiñan et al. 2023). However, this effect is markedly asymmetric across tumour types.

While *HLTF* expression is significantly increased in 19 of 25 cancer types with copy-number gain (Supplementary Table S9), a statistically significant decrease upon heterozygous loss is detected only in 4 of 19 cancer types (Supplementary Table S8). This asymmetric response suggests that *HLTF* may operate under a dosage-sufficiency model, whereby a single functional allele is sufficient to maintain basal expression (Bravo-Estupiñan et al. 2023). Alternatively, compensatory regulatory mechanisms could mitigate the effects of copy loss.

In uveal melanoma (UVM) single-copy deletions of *HLTF* are significantly associated with decreased overall survival (OS) (Fig. 2C; Supplementary Table S14). Nevertheless, since large-scale deletions and loss of chromosome 3 are frequently observed in this cancer type, the specific contribution of *HLTF* deletion cannot be evaluated in isolation (Tschentscher et al. 2001). Indeed, comparable patterns of single copy deletions can be observed across all neighbouring loci, consistent with broad chromosomal alterations rather than gene-specific effects (Supplementary Figure S4).

### Rare *HLTF* loss-of-function mutations are enriched in UCEC and associate with outcome

**Fig. 3:**
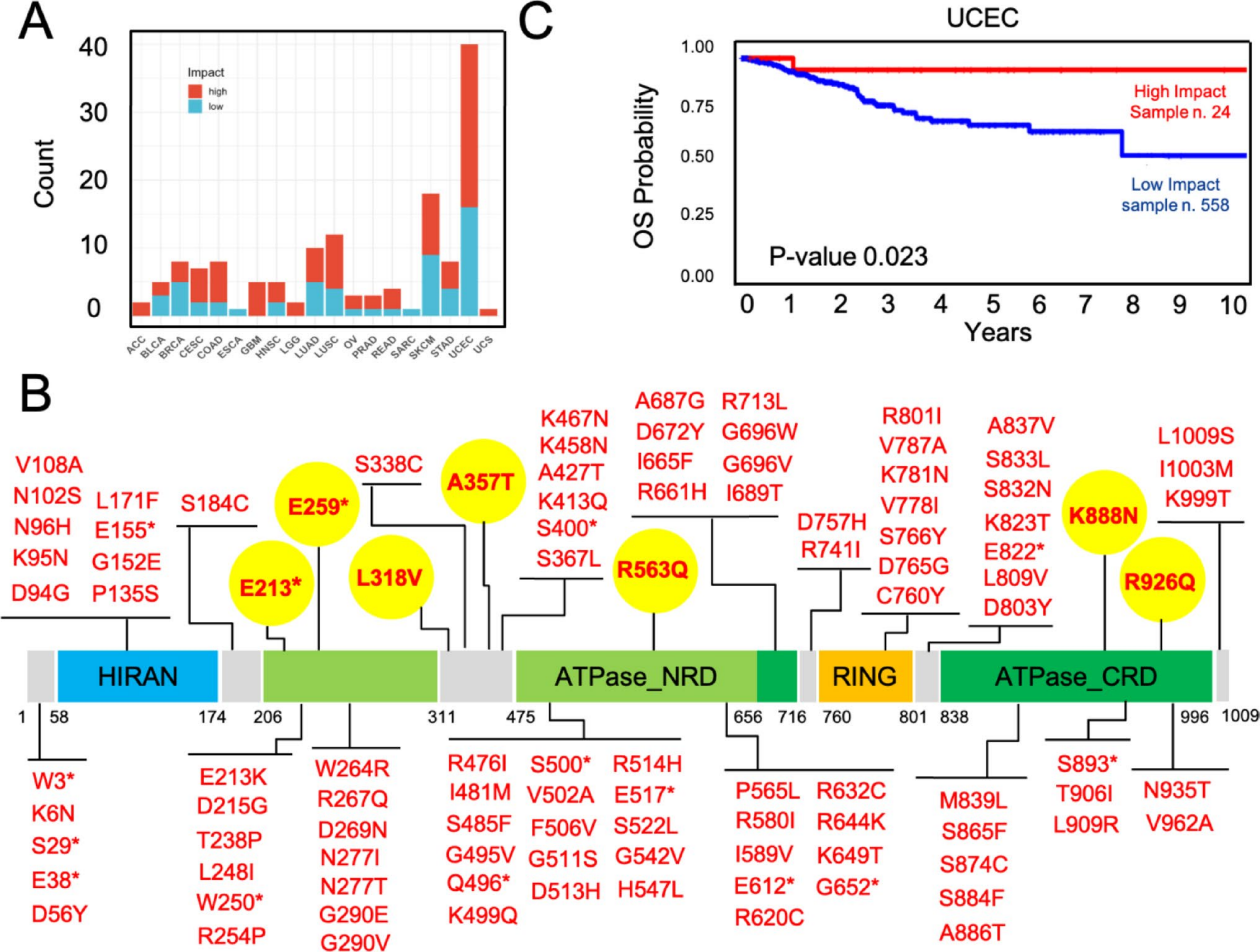
**A** Distribution of *HLTF* mutations with high- (red) and low- (blue) impact across TCGA cancer types. Each bar plot shows the number of *HLTF* somatic variants defined as described in Supplementary Table S5. Most tumour types display a predominance of low-impact *HLTF* variants, except UCEC which shows the highest number of deleterious *HLTF* mutations. **B** The predicted high-impact somatic mutations (in red) on HLTF protein domain architecture: HIRAN, ATPase motor-remodeling N- and C- lobes, and RING. The 7 mutations found in at least two distinct cancer types are highlighted in bold and circled by a yellow sphere. Some of the remaining can occur together: S184C+ K781N; N277I+ A427T+C760Y+S865F; R267Q +V502A; K467N+R476I; R563Q+A886T; S766Y+R926Q; E213K+K499Q+M839L; N96H+D803Y+R926Q; D269N+K458N; E38*+R563Q+L809V; E517*+R801I; L171F+K413Q; K95N+K823T; E213*+A357T+V778I+E822*+T906I; E612*+V787A+K888N; K6N+E213*; E259*+R741I; D56Y+K888N; R580I+V962A; F506V+K888N. **C** Kaplan–Meier plot of Overall Survival (OS) extracted and stratified by *HLTF* high- (red) and low- (blue) impact mutations in UCEC; sample numbers and the overall p-value are reported

According to publicly available data, somatic mutations affecting *HLTF* were infrequent in the TCGA PanCancer cohort. Using cBioPortal, we retrieved 178 *HLTF* somatic mutations in 143 distinct cancer patients (Supplementary Table S11), consistent with previous reports of low HLTF mutation frequency in cancer (Dhont et al. 2018). Based on functional impact annotation, 86 mutations were classified as deleterious according to our criteria (Fig. 3A; Supplementary Table S11). The relative proportion of predicted LoF somatic mutations in *HLTF* were broadly comparable, with no statistically significant difference across cancer types (Supplementary Table S10), and nine somatic mutations were independently detected in at least two distinct cancer types/patients. Among these recurrent events seven had a deleterious impact on protein function, namely E213*, E259* L318V, A357T, R563Q, K888N, and R926Q, and were primarily located within the ATPase motor/remodelling domain (Ljubic et al. 2024) (Fig. 3B), whereas the remaining somatic mutations distributed evenly across the entire protein sequence (Fig. 3B). Interestingly, five of the seven *HLTF* variants identified in the TCGA/cBioPortal somatic mutation dataset - E259*, A357T, R563Q, K888N, and R926Q - are also reported in gnomAD at very low population frequencies (< 1 × 10^− 5^). Although the presence of a variant in gnomAD does not by itself imply that the corresponding TCGA alteration is germline or incorrectly annotated, we cannot formally exclude residual misclassification of rare germline variants in publicly available somatic mutation calls. Therefore, individual rare *HLTF* variants should be interpreted with caution, particularly when assigning functional or clinical significance. At the same time, such overlap with population databases does not rule out the possibility that these variants may also arise as acquired somatic events or be subject to tumour-associated selection.

Notably, uterine corpus endometrial carcinoma (UCEC) and Skin Cutaneous Melanoma (SKCM) displayed an increased burden of HLTF somatic mutations compared to other cancer types (UCEC: OR = 6.8, FDR ≤ 2e-16; SKCM: OR = 2.18, FDR = 0.03) (Supplementary Table S10). Notably, in UCEC predicted deleterious/LoF variants in *HLTF* were also associated with improved overall survival (Fig. 3C), suggesting that reduced *HLTF* function may confer a favourable prognostic effect in a tumour-specific context.

## Discussion

This pan‑cancer, multi‑omics analysis delineated a complex landscape of *HLTF* alterations across human tumours. By integrating mRNA expression, DNA methylation at promoter‑proximal and promoter‑distal elements, copy‑number alterations (CNAs) and somatic loss‑of‑function (LoF) mutations, we show that *HLTF* function can be perturbed through multiple, partially independent mechanisms whose phenotypic and prognostic consequences vary by tumour type.

At the transcriptional level, *HLTF* mRNA is both up- and down-regulated across cancers. Notably, the direction of dysregulation does not translate into a uniform prognostic outcome: in some cancer types, higher expression associates with poorer survival, whereas in others it does not, and also the inverse pattern can be observed. This heterogeneity is consistent with a dual‑agent role in which *HLTF* can either restrain or promote tumorigenesis depending on the molecular and cellular context. Mechanistically, this may reflect (i) the reliance on *HLTF*’s roles in DNA-damage tolerance and replication-stress response versus its transcriptional regulation, (ii) tumour-specific regulatory architecture (promoter/enhancer usage, transcription‑factor availability), and (iii) microenvironmental composition. In kidney cancers, for example, *HLTF* expression in KIRC correlates with CD4⁺ T‑cell - but not CD8A - signatures, whereas no pattern can be detected in KIRP (Supplementary Figure S5), suggesting tumour‑specific coupling to immune infiltration pathways that may modulate the net impact of *HLTF* expression on disease behaviour (Govindarajan et al. 2023). A similar subtype-dependent response in tumours was also reported for SMARCAL1, a related SWI/SNF-associated factor (Leuzzi et al. 2024; Zhao et al. 2025).

*HLTF* promoter hypermethylation has long been implicated in gastrointestinal carcinogenesis (Moinova et al. 2002; Hamai et al. 2003; Liu et al. 2018). Consistently, we identified cancers with increased promoter methylation accompanied by reduced *HLTF* expression, and we confirmed this relationship in COAD, READ and STAD. In contrast to reports linking promoter hypermethylation and poor prognosis reported in selected cohorts, our survival analyses did not detect significant associations between reduced *HLTF* expression and decreased survival in COAD, STAD and READ. This discrepancy may reflect limited statistical power and cohort-specific clinical composition, and warrants validation in larger and clinically stratified datasets. Notably, we also uncovered similar promoter methylation-expression patterns in other cancer types (HNSC, ESCA, and DLBCL), suggesting that epigenetic silencing of *HLTF* may be more pervasive than previously appreciated. Beyond the promoter, analysis of a candidate *HLTF* enhancer ~1 kb upstream of the TSS revealed modest negative tissue-specific associations between *HLTF* methylation and its expression (Supplementary Figure S3). Reduced methylation at this distal site coincides with elevated *HLTF* mRNA levels, consistent with enhancer activation. Yet, this effect is not uniform, being evident in LUAD and CESC, weaker in SARC, and absent in STAD and TGCT, indicating tissue-specific regulatory control likely shaped by chromatin context and transcription-factor availability (Jones and Baylin 2007; Smith et al. 2020). Functional follow‑up, including reporter assays, CRISPRi/CRISPRa targeting of the distal element, and ChIP‑seq for candidate transcription factors, will be required to establish causality and context dependency.

Across the pan‑cancer cohort, CNAs at the *HLTF* locus are relatively common, with only ~ 60% of the samples retaining a diploid copy number. Copy-number gains were associated with proportional increases in *HLTF* mRNA, whereas heterozygous loss tended to reduce expression relative to diploid. Interestingly, the magnitude of decreased expression after heterozygous loss is attenuated, consistent with a dosage-sufficiency model in which one allele maintains near-baseline transcription, potentially buffered by compensatory regulatory mechanisms (Hose et al. 2015). Uveal melanoma (UVM) illustrates both the opportunity and the caveat of CNA analyses: while decreased survival was observed in the group with copy loss, arm‑level or chromosome‑wide events (frequent chromosome 3 loss) are common in UVM, preventing attribution of this association to *HLTF* in isolation. Indeed, neighbouring genes showed comparable deletion patterns, pointing to broad chromosomal alterations rather than gene‑specific effects.

At the sequence level, *HLTF* shows an excess of somatic mutations in SKCM and UCEC, with the latter showing high‑impact variants comprising a majority of observed mutations. Intriguingly, patients harbouring these LoF variants experienced better survival than those with neutral‑impact mutations suggesting that reduced *HLTF* function may confer a favourable effect in a tumour-specific context.

Taken together, these findings outline a multifaceted and context‑contingent regulatory landscape. In some settings, particularly where promoter hypermethylation silences *HLTF*, loss of expression is compatible with tumour‑suppressive functions (e.g., genome maintenance). In others, enhancer‑driven up‑regulation or dosage gains may couple *HLTF* to transcriptional programmes that facilitate growth or immune‑microenvironment remodelling, leading to neutral or even favourable prognostic associations for decreased expression or LoF in specific contexts (e.g., UCEC/MSI‑H). Overall, lineage, regulatory architecture (promoter versus distal control), co-alterations (arm-level CNAs, mismatch-repair deficiency) and microenvironmental composition likely converge to determine whether *HLTF* acts as a tumour suppressor or as an oncogenic facilitator, consistent with a “double-agent” model (Shen et al. 2018). However, because tumour purity estimates were not included in our analyses of *HLTF* methylation and expression, future studies aimed at validating these findings could account for tumour purity as a potential confounder. This aspect is particularly relevant for cancer types such as PAAD, which are characterized by relatively low tumour purity.

## Conclusions

Despite the need of (i) perturbation experiments to directly map causal relationships between *HLTF* regulatory regions and (ii) additional samples for both tumour-subtype- and CNA- associated survival analyses, this integrative work across TCGA cancers revealed that *HLTF* alterome is diversified and context‑dependent, encompassing promoter and enhancer methylation, impactful CNAs with lineage‑specific dosage responses, and loss-of-function mutations with context‑specific survival associations.

Altogether, this information supports a yet uncharacterized “double-agent” mode in which *HLTF*’s net effect on tumour behaviour and prognosis varies across tissues and molecular backgrounds, thus underlying its biomarker potential that extend to prognostic relevance particularly in UCEC.

## Supplementary Information

Below is the link to the electronic supplementary material.

Supplementary material 1 (DOCX 5134.5 kb)

## Data Availability

Publicity available data were retrieved from The Cancer Genome Atlas (TCGA).
