# Supplementary material for: Multi-omics analysis of HLTF context-dependent regulation across TCGA cohorts revealed its potential as new biomarker in tumour
Source: Mamm Genome. 2026 Jul 24;37(1):92. doi: 10.1007/s00335-026-10257-w (PMC13400721; doi:10.1007/s00335-026-10257-w)
Supplement: Supplementary file 1 — Supplementary material 1 (DOCX 5134.5 kb) [file 335_2026_10257_MOESM1_ESM.docx]

**Supplementary Information**

**Multi-omics analysis of *HLTF* context-dependent regulation across TCGA cohorts revealed its potential as new biomarker in tumour**

Alessia Canevotti ^1,2^, Marija Tursunović ^3^, Wendalina Tigani ^4^, Graziano Pesole ^2,5^, Matteo Chiara ^1*^ and Matteo De March ^6*^

^1^ Dipartimento di Bioscienze, Università degli Studi di Milano Statale, via Celoria 26, 20133 Milano, Italy

^2^ SEMM, European School of Molecular Medicine, Milan 20139, Italy

^3^ Innovative Centre of the Faculty of Chemistry, University of Belgrade, 11158 Belgrade, Serbia

^4^ Department of Life Sciences, University of Trieste, via Licio Giorgieri 5, 34100, Trieste, Italy

^5^ Dipartimento di Bioscienze, Biotecnologie E Ambiente, Università degli Studi di Bari Aldo Moro, Piazza Umberto I, 70121 Bari, Italy

^6^ Laboratory for Environmental and Life Sciences, University of Nova Gorica, Vipaska 13, SI-5000, Nova Gorica, Slovenia

This file includes **Supplementary Tables S1-S14**, and **Supplementary Figures S1-S5**.

| **Cancer**  **type** | **mRNA** | **Methylation** | | | | **CNAs** | **Mutations** |
| --- | --- | --- | --- | --- | --- | --- | --- |
|  |  | **cg26151310** | **cg04836786** | **cg02059813** | **cg21089667** |  |  |
| **ACC** | 75 | NA | NA | NA | NA | 90 | 92 |
| **BLCA** | 403 | 180 | NA | 180 | 180 | 408 | 411 |
| **BRCA** | 1074 | 523 | 523 | 523 | 523 | 1080 | 792 |
| **CESC** | 292 | NA | 81 | 81 | 81 | 295 | 290 |
| **CHOL** | 36 | NA | NA | 24 | NA | 36 | NA |
| **COAD** | 289 | 206 | 207 | 206 | 206 | 451 | 295 |
| **DLBCL** | 47 | 29 | 29 | 29 | 29 | 48 | NA |
| **ESCA** | 180 | 57 | 57 | 57 | 57 | 184 | 184 |
| **GBM** | 147 | 461 | 38 | NA | NA | 577 | 315 |
| **HNSC** | 512 | 150 | 161 | 161 | 161 | 522 | 508 |
| **KICH** | 66 | NA | NA | NA | 51 | 66 | NA |
| **KIRC** | 522 | NA | 217 | 217 | 217 | 528 | NA |
| **KIRP** | 286 | 169 | 169 | 169 | 169 | 288 | NA |
| **LAML** | 165 | NA | NA | 159 | 159 | 191 | NA |
| **LGG** | 506 | NA | 461 | 461 | 461 | 513 | 515 |
| **LIHC** | 362 | 218 | 260 | 260 | 260 | 370 | NA |
| **LUAD** | 510 | 265 | 233 | 233 | 233 | 516 | 518 |
| **LUSC** | 495 | 229 | 54 | NA | NA | 501 | 481 |
| **MESO** | 87 | NA | 63 | NA | 63 | 87 | NA |
| **OV** | 415 | NA | NA | NA | NA | 579 | 630 |
| **PAAD** | 177 | NA | 132 | 132 | 132 | 184 | NA |
| **PCPG** | 161 | NA | NA | 68 | 68 | 162 | NA |
| **PRAD** | 489 | NA | NA | 403 | 403 | 492 | 495 |
| **READ** | 91 | 66 | 57 | 57 | 57 | 165 | 94 |
| **SARC** | 254 | 133 | 156 | 156 | 156 | 257 | 236 |
| **SKCM** | 365 | 404 | 235 | NA | 235 | 367 | 466 |
| **STAD** | 412 | 230 | 230 | 230 | 230 | 441 | 440 |
| **TGCT** | 148 | NA | 85 | 85 | 85 | 150 | NA |
| **THCA** | 496 | 235 | 488 | 488 | 488 | 499 | NA |
| **THYM** | 118 | 158 | 106 | 106 | 106 | 123 | NA |
| **UCEC** | 180 | 186 | 110 | 110 | 110 | 539 | 472 |
| **UCS** | 56 | NA | NA | 20 | NA | 56 | 58 |
| **UVM** | 79 | NA | NA | NA | 36 | 80 | NA |

**Supplementary Table S1.** For each cancer type and *HLTF* omics feature (mRNA expression, methylation status mapped by the 4 probes, CNAs, and somatic mutations) the number of samples retrieved from TCGA PanCancer Atlas [Goldman et al. 2020: doi:10.1038/s41587-020-0546-8. Gao et al. 2013: doi:10.1126/scisignal.2004088. Cerami et al. 2012: doi:10.1158/2159-8290.CD-12-0095] are displayed.

| **Cancer**  **type** | **Tumour count** | **Normal count** | **p-value**  **(ks test)** | **LogFC** | **Correlation between low**  **expression**  **and OS** | **Correlation between high**  **expression**  **and OS** |
| --- | --- | --- | --- | --- | --- | --- |
| **BLCA** | 19 | 19 | 0.3 | 0.71 | Not significant | Not significant |
| **BRCA*** | 112 | 112 | 1.24e^-08 | 0.6 | Not significant | Not significant |
| **CHOL*** | 9 | 9 | 0.00074 | 1.39 | Not significant | Not significant |
| **COAD** | 26 | 26 | 0.085 | -0.02 | Not significant | Not significant |
| **ESCA*** | 13 | 13 | 0.044 | 1.46 | Not significant | Not significant |
| **HNSC*** | 43 | 43 | 1.36e^-09 | 1.33 | Not significant | Not significant |
| **KICH*** | 25 | 25 | 0.002 | -0.72 | Increased survival | Decreased survival |
| **KIRC*** | 72 | 72 | 1.2e^-05 | -0.5 | Decreased survival | Increased survival |
| **KIRP*** | 32 | 32 | 0.004 | -0.47 | Not significant | Not significant |
| **LIHC*** | 50 | 50 | 2.6e^-09 | 1.14 | Increased survival | Decreased survival |
| **LUAD*** | 58 | 58 | 5.6e^-08 | 0.84 | Not significant | Not significant |
| **LUSC*** | 50 | 50 | 2.7e^-11 | 1.32 | Not significant | Not significant |
| **PRAD** | 52 | 52 | 0.045 | 0.4 | Not significant | Not significant |
| **STAD** | 33 | 33 | 0.65 | 0.4 | Not significant | Not significant |
| **THCA*** | 59 | 59 | 1.4e^-10 | -0.82 | Not significant | Not significant |

**Supplementary Table S2.** Differential expression of *HLTF* between tumor and matched normal tissues across TCGA cancer types. The table reports the number of paired tumor and normal samples analyzed, together with the p-values extracted from the Kolmogorov–Smirnov (KS) test [Massey et al. 1951: doi.org/10.1080/01621459.1951.10500769] and Log₂(FC) of *HLTF* expression. All cancer types with at least 9 available tumors–normal matched pairs are included; statistical significance is attributed with p < 0.05. The “Low expression” and “High expression” columns indicate the correlation between HLTF expression and overall survival (OS), when statistically significant.

|  | **cg26151310** | | | | **cg04836786** | | | |
| --- | --- | --- | --- | --- | --- | --- | --- | --- |
| **Cancer type** | **n. of samples**  **β≥0.1 (non-hypomethylated)** | **n. of samples β<0.1 (hypomethylated)** | **p-value** | **FDR** | **n. of samples β≥0.1 (non-hypomethylated)** | **n. of samples β<0.1 (hypomethylated)** | **p-value** | **FDR** |
| **BLCA** | 1 | 179 | 1 | 1 | - | - | - | NA |
| **BRCA** | 3 | 520 | 1 | 1 | 6 | 517 | 1 | 1.00 |
| **CESC** | - | - | - | NA | 1 | 80 | 1.27 | 1.00 |
| **COAD** | 60 | 146 | 2.2e^-50 | 9.0e^-16 | 92 | 113 | 2.6e^-103 | 7.67e^-16 |
| **DLBCL** | 4 | 25 | 0.05 | 1.8e^-02 | 5 | 24 | 6.2e^-04 | 2.85e^-03 |
| **ESCA** | 5 | 52 | 0.01 | 3.0e^-02 | 6 | 51 | 2.6e^-03 | 9.97e^-03 |
| **GBM** | 1 | 460 | 1 | 1 | 1 | 37 | 1.01 | 0.891 |
| **HNSC** | 11 | 150 | 0.02 | 9.0e^-03 | 27 | 134 | 1e^-15 | 5.75e^-15 |
| **KIRC** | - | - | - | NA | 5 | 212 | 1.02 | 0.891 |
| **KIRP** | 4 | 165 | 0.6 | 0.98 | 6 | 163 | 0.23 | 0.588 |
| **LGG** | - | - | - | NA | 2 | 459 | 1 | 1.00 |
| **LIHC** | 1 | 217 | 1 | 1 | 7 | 253 | 0.46 | 0.882 |
| **LUAD** | 6 | 259 | 0.5 | 0.9 | 8 | 225 | 0.21 | 0.588 |
| **LUSC** | 2 | 227 | 0.4 | 0.9 | 2 | 52 | 0.38 | 0.857 |
| **MESO** | - | - | - | NA | 1 | 62 | 1.19 | 1.00 |
| **PAAD** | - | - | - | NA | 4 | 128 | 0.41 | 0.857 |
| **READ** | 14 | 52 | 5.3e^-11 | 3.18e^-10 | 21 | 36 | 4.7e^-20 | 7.67e^-16 |
| **SARC** | 1 | 132 | 1 | 1 | 4 | 152 | 0.54 | 0.887 |
| **SKCM** | 1 | 403 | 1 | 1 | 3 | 232 | 1.33 | 1.00 |
| **STAD** | 47 | 183 | 2.8e^-31 | 9.0e^-16 | 64 | 166 | 1.4e^-52 | 7.67e^-16 |
| **TGCT** | - | - | - | NA | 5 | 80 | 0.57 | 0.187 |
| **THCA** | 1 | 234 | 1 | 1 | 2 | 486 | 1 | 1.00 |
| **THYM** | 3 | 155 | 0.5 | 0.9 | 2 | 104 | 1.13 | 0.988 |
| **UCEC** | 3 | 183 | 0.5 | 0.9 | 3 | 107 | 0.5 | 0.885 |

**Supplementary Table S3.** HLTF methylation extracted from the promoter-proximal probes. For each cancer type with at least one available data point, the number of samples with methylation values ≥0.1 (non-hypomethylated) and <0.1 (hypomethylated) are reported along with the p-values indicating the enrichment in hypomethylated samples. False Discovery Rates (FDR) adjusted for multiple testing are also provided.

|  | **cg21089667** | | | | **cg02059813** | | | |
| --- | --- | --- | --- | --- | --- | --- | --- | --- |
| **Cancer type** | **n. of samples**  **β≤0.9 (non-hypermethylated)** | **n. of samples**  **β>0.9 (hypermethylated)** | **p-value** | **FDR** | **n. of samples**  **β≤0.9 (non-hypermethylated)** | **n. of samples**  **β>0.9 (hypermethylated)** | **p-value** | **FDR** |
| **AML** | 4 | 155 | 1 | 1.00 | 1 | 158 | 1 | 1.00 |
| **BLCA** | 22 | 158 | 1 | 1.00 | 4 | 176 | 1 | 1.00 |
| **BRCA** | 18 | 505 | 1 | 1.00 | 14 | 509 | 1 | 1.00 |
| **CESC** | 27 | 54 | 2e^-08 | 1.93e^-07 | 18 | 63 | 2e^-08 | 1.67e^-07 |
| **CHOL** | – | – | – | NA | 1 | 23 | 0.6 | 1.00 |
| **COAD** | 24 | 182 | 1 | 1.00 | 5 | 201 | 1 | 1.00 |
| **DLBCL** | 4 | 25 | 0.15 | 0.725 | 3 | 26 | 0.15 | 0.625 |
| **ESCA** | 12 | 45 | 0.75 | 1.00 | 2 | 55 | 0.76 | 1.00 |
| **HNSC** | 29 | 132 | 0.77 | 1.00 | 6 | 155 | 0.77 | 1.00 |
| **KICH** | 7 | 44 | 1 | 1.00 | – | – | – | NA |
| **KIRC** | 4 | 213 | 1 | 1.00 | 4 | 213 | 1 | 1.00 |
| **KIRP** | 8 | 161 | 1 | 1.00 | 2 | 167 | 1 | 1.00 |
| **LGG** | 21 | 440 | 1 | 1.00 | 1 | 460 | 1 | 1.00 |
| **LIHC** | 27 | 233 | 1 | 1.00 | 1 | 259 | 1 | 1.00 |
| **LUAD** | 188 | 45 | 2.8e^-41 | 1.45e^-15 | 71 | 162 | 2.7e^-41 | 1.25e^-15 |
| **LUSC** | 5 | 49 | 1 | 1.00 | – | – | – | NA |
| **MESO** | 5 | 58 | 1 | 1.00 | – | – | – | NA |
| **PAAD** | 14 | 118 | 1 | 1.00 | 2 | 130 | 1 | 1.00 |
| **PCPG** | 1 | 67 | 1 | 1.00 | 1 | 67 | 1 | 1.00 |
| **PRAD** | 19 | 384 | 1 | 1.00 | 1 | 402 | 1 | 1.00 |
| **READ** | 6 | 51 | 0.75 | 1.00 | 2 | 55 | 0.75 | 1.00 |
| **SARC** | 16 | 140 | 2.4e^-03 | 1.39e^-02 | 16 | 140 | 2e^-03 | 1.00e^-02 |
| **SKCM** | 18 | 217 | 1 | 1.00 | – | – | – | NA |
| **STAD** | 39 | 191 | 7e^-06 | 5.08e^-05 | 27 | 203 | 7e^-06 | 4.38e^-05 |
| **TGCT** | 59 | 26 | 1.6e^-44 | 1.45e^-15 | 49 | 36 | 1.6e^-44 | 1.25e^-15 |
| **THCA** | 19 | 469 | 1 | 1.00 | 4 | 484 | 1 | 1.00 |
| **THYM** | 5 | 101 | 1 | 1.00 | 1 | 105 | 1 | 1.00 |
| **UCEC** | 7 | 103 | 0.89 | 1.00 | 3 | 107 | 1 | 1.00 |
| **UCS** | 4 | 16 | 0.24 | 0.994 | 2 | 18 | 0.2 | 0.714 |
| **UVM** | 2 | 34 | 1 | 1.00 | – | – | – | NA |

**Supplementary Table S4.** *HLTF* methylation extracted from the promoter-distal probes. For each cancer type with at least one available data point the number of samples with methylation values ≤0.9 (non-hypermethylated) and > 0.9 (hypermethylated) are reported along with the p-values indicating the enrichment in non-hypermethylated samples. False Discovery Rates (FDR) adjusted for multiple testing are also provided.

| **Probe** | **Pearson r** | **p-value** | **Slope** |
| --- | --- | --- | --- |
| **cg26151310** | -0.31 | <2e^-16 | -0.01 |
| **cg04836786** | -0.38 | <2e^-16 | -0.03 |
| **cg21089667** | 0.008 | 5.85e^-01 | 0 |
| **cg02059813** | -0.03 | 1.88e^-02 | 0 |

**Supplementary Table S5.** Correlation between *HLTF* expression and CpG methylation levels. The table reports Pearson correlation coefficients (r), corresponding p-values, and regression slopes for the four Illumina HumanMethylation450 array probes cg26151310, cg04836786, cg21089667 and cg02059813.

| **Cancer type** | **HomDel** | **HetLoss** | **Diploid** | **Gain** | **Amp** |
| --- | --- | --- | --- | --- | --- |
| **ACC** | 0 | 19 | 51 | 19 | 1 |
| **BLCA** | 0 | 16 | 182 | 197 | 13 |
| **BRCA** | 0 | 74 | 711 | 272 | 23 |
| **CESC** | 0 | 6 | 82 | 171 | 36 |
| **CHOL** | 0 | 4 | 24 | 8 | 0 |
| **COAD** | 0 | 34 | 350 | 67 | 0 |
| **DLBCL** | 1 | 4 | 30 | 11 | 2 |
| **ESCA** | 1 | 12 | 59 | 85 | 27 |
| **HNSC** | 0 | 9 | 165 | 274 | 74 |
| **GBM** | 3 | 40 | 463 | 68 | 3 |
| **KICH** | 0 | 8 | 51 | 7 | 0 |
| **KIRC** | 0 | 73 | 377 | 74 | 4 |
| **KIRP** | 0 | 8 | 184 | 96 | 0 |
| **LAML** | 0 | 3 | 187 | 1 | 0 |
| **LGG** | 1 | 35 | 469 | 8 | 0 |
| **LIHC** | 0 | 40 | 270 | 55 | 5 |
| **LUAD** | 1 | 116 | 269 | 119 | 11 |
| **LUSC** | 1 | 15 | 68 | 286 | 131 |
| **MESO** | 1 | 5 | 63 | 17 | 1 |
| **OV** | 0 | 21 | 163 | 315 | 80 |
| **PAAD** | 0 | 19 | 139 | 24 | 2 |
| **PCPG** | 0 | 93 | 69 | 0 | 0 |
| **PRAD** | 4 | 8 | 409 | 62 | 9 |
| **READ** | 0 | 10 | 118 | 37 | 0 |
| **SARC** | 1 | 50 | 160 | 43 | 3 |
| **SKCM** | 3 | 55 | 237 | 70 | 2 |
| **STAD** | 0 | 37 | 281 | 114 | 9 |
| **TGCT** | 0 | 20 | 87 | 43 | 0 |
| **THYM** | 0 | 7 | 111 | 5 | 0 |
| **THCA** | 0 | 4 | 493 | 2 | 0 |
| **UCS** | 0 | 7 | 20 | 27 | 2 |
| **UCEC** | 1 | 17 | 395 | 105 | 21 |
| **UVM** | 0 | 44 | 36 | 0 | 0 |

**Supplementary Table S6.** *HLTF* CNAs and sample count across cancer types as defined in the cBioPortal. HomDel: deletion of both copies of the gene. HetLoss: deletion of one copy gene. Diploid: no CNA. Gain: acquisition of a single extra copy of the gene. Amp: acquisition of multiple extra copies of the gene.

| **Cancer type** | **p-value** | **FDR** |
| --- | --- | --- |
| **ACC** | 0.15 | 0.412 |
| **BLCA** | 7e-14 | 3.85e-13 |
| **BRCA** | 1 | 1 |
| **CESC** | 1.7e-34 | 1.4025e-33 |
| **CHOL** | 0.75 | 1 |
| **COAD** | 1 | 1 |
| **DLBC** | 0.56 | 1 |
| **ESCA** | 3.6e-17 | 2.376e-16 |
| **GBM** | 1 | 1 |
| **HNSC** | 1.6e-48 | 1.76e-47 |
| **KICH** | 1 | 1 |
| **KIRC** | 1 | 1 |
| **KIRP** | 0.7 | 1 |
| **LAML** | 1 | 1 |
| **LGG** | 1 | 1 |
| **LIHC** | 1 | 1 |
| **LUAD** | 6e-07 | 2.475e-06 |
| **LUSC** | 4.5e-119 | 1.485e-117 |
| **MESO** | 0.98 | 1 |
| **OV** | 1.8e-66 | 2.97e-65 |
| **PAAD** | 1 | 1 |
| **PCPG** | 1.8e-07 | 8.4e-07 |
| **PRAD** | 1 | 1 |
| **READ** | 1 | 1 |
| **SARC** | 0.5 | 1 |
| **SKCM** | 0.82 | 1 |
| **STAD** | 0.72 | 1 |
| **TGCT** | 0.15 | 0.412 |
| **THCA** | 1 | 1 |
| **THYM** | 1 | 1 |
| **UCEC** | 1 | 1 |
| **UCS** | 4e-05 | 0.00014 |
| **UVM** | 0.001 | 0.0033 |

**Supplementary Table S7.** Overall enrichment of any CNAs compared with Diploid condition across all cancer types for which data are available. The p-values and the adjusted p-value (FDR) are indicated. A significant FDR (<0.05) indicates a statistically significant increase in the proportion of CNAs in a cancer type, compared to the background of all the other cancer types.

| **Cancer type** | **Log_2_FC HetLoss** | **FDR** |
| --- | --- | --- |
| **BRCA** | -0.146 | 0.012 |
| **LUAD** | -0.157 | 0.043 |
| **PCPG** | -0.369 | 3.35E-08 |
| **SKCM** | -0.352 | 0.0004 |

**Supplementary Table S8.** Cancers with a statistically significant decrease in *HLTF* expression following heterozygous copy loss (HetLoss).

| **Cancer type** | **Log_2_FC gain** | **FDR** |
| --- | --- | --- |
| **ACC** | 0.476 | 0.026 |
| **BLCA** | 0.340 | 6.70E-11 |
| **BRCA** | 0.105 | 8.99E-07 |
| **CESC** | 0.181 | 0.006 |
| **COAD** | 0.331 | 0.01 |
| **ESCA** | 0.404 | 9.21E-05 |
| **HNSC** | 0.634 | 5.98E-12 |
| **KIRP** | 0.165 | 0.026 |
| **LIHC** | 0.256 | 0.006 |
| **LUAD** | 0.308 | 1.68E-14 |
| **LUSC** | 0.250 | 6.53E-07 |
| **OV** | 0.271 | 3.16E-08 |
| **PAAD** | 0.206 | 0.019 |
| **PRAD** | 0.186 | 0.001 |
| **SARC** | 0.235 | 0.004 |
| **SKCM** | 0.074 | 0.046 |
| **STAD** | 0.267 | 0.0002 |
| **UCEC** | 0.186 | 0.033 |
| **UCS** | 0.323 | 0.008 |

**Supplementary Table S9.** Cancers with a statistically significant increase in *HLTF* expression following heterozygous copy gain (Gain).

| **Cancer type** | **With HLTF somatic mutations** | **Without HLTF somatic mutations** | **OR** | **p-value** | **FDR** |
| --- | --- | --- | --- | --- | --- |
| ACC | 2 | 88 | 1.11 | 0.54 | 1.00 |
| BLCA | 5 | 401 | 0.59 | 0.92 | 1.00 |
| BRCA | 8 | 776 | 0.47 | 0.99 | 1.00 |
| CESC | 7 | 275 | 1.25 | 0.34 | 1.00 |
| COAD | 8 | 275 | 1.44 | 0.21 | 0.88 |
| ESCA | 1 | 182 | 0.26 | 0.98 | 1.00 |
| GBM | 5 | 305 | 0.79 | 0.75 | 1.00 |
| HNSC | 5 | 497 | 0.47 | 0.98 | 1.00 |
| LGG | 2 | 507 | 0.18 | 1.00 | 1.00 |
| LUAD | 10 | 496 | 0.98 | 0.57 | 1.00 |
| LUSC | 12 | 457 | 1.31 | 0.23 | 0.88 |
| OV | 3 | 624 | 0.22 | 1.00 | 1.00 |
| PRAD | 3 | 489 | 0.28 | 1.00 | 1.00 |
| READ | 4 | 83 | 2.39 | 0.10 | 0.62 |
| SARC | 1 | 234 | 0.20 | 0.99 | 1.00 |
| SKCM | 18 | 430 | 2.19 | 0.0034 | 0.03 |
| STAD | 8 | 423 | 0.92 | 0.64 | 1.00 |
| UCEC | 40 | 373 | 6.86 | 2e-17 | 2e-16 |
| UCS | 1 | 55 | 0.89 | 0.68 | 1.00 |

**Supplementary Table S10.** Cancer types enriched in samples with somatic *HLTF* mutations for each cancer type, the number of samples with detected somatic *HLTF* mutations and samples without somatic mutations in *HLTF* is reported. Enrichment was assessed by comparing the proportion of mutated samples in each tumor type with that observed in all other tumors using a one-sided Fisher’s exact test. Odds ratios (OR), p-values, and False Discovery Rate (FDR; Benjamini–Hochberg correction) are shown.

| **Sample ID** | **Cancer type** | **Protein change** | **Impact** |
| --- | --- | --- | --- |
| TCGA-02-0033-01 | **GBM** | W3* | high |
| TCGA-05-4382-01 | **LUAD** | K781N, S184C | high |
| TCGA-05-4427-01 | **LUAD** | H547L | high |
| TCGA-06-0171-01 | **GBM** | S29* | high |
| TCGA-06-0171-02 | **GBM** | S29* | high |
| TCGA-06-5416-01 | **GBM** | N277T | high |
| TCGA-17-Z011-01 | **LUAD** | Q15H | low |
| TCGA-17-Z015-01 | **LUAD** | R632C | high |
| TCGA-17-Z017-01 | **LUAD** | T987I | low |
| TCGA-18-4083-01 | **LUSC** | T958I | high |
| TCGA-21-1080-01 | **LUSC** | G290E | high |
| TCGA-21-1081-01 | **LUSC** | A818T | low |
| TCGA-25-1313-01 | **OV** | S367L | high |
| TCGA-29-1696-01 | **OV** | N24S | low |
| TCGA-2A-A8VT-01 | **PRAD** | I589V | high |
| TCGA-2W-A8YY-01 | **CESC** | R384H, N899Tfs*9 | low |
| TCGA-37-4130-01 | **LUSC** | X299_splice | low |
| TCGA-55-8204-01 | **LUAD** | K976R, N532S | low |
| TCGA-55-8205-01 | **LUAD** | D757H | high |
| TCGA-60-2698-01 | **LUSC** | L318V | high |
| TCGA-60-2714-01 | **LUSC** | V21F | low |
| TCGA-61-2096-01 | **OV** | D765G | high |
| TCGA-66-2754-01 | **LUSC** | W250* | high |
| TCGA-66-2786-01 | **LUSC** | A725S | low |
| TCGA-75-5126-01 | **LUAD** | X177_splice | low |
| TCGA-76-6285-01 | **GBM** | G511S | high |
| TCGA-85-8070-01 | **LUSC** | G495V | high |
| TCGA-85-8277-01 | **LUSC** | R254P | high |
| TCGA-86-8278-01 | **LUAD** | L63F | low |
| TCGA-98-7454-01 | **LUSC** | G290V | high |
| TCGA-98-8021-01 | **LUSC** | S522L | high |
| TCGA-A2-A0T1-01 | **BRCA** | I1000M | low |
| TCGA-A2-A0YK-01 | **BRCA** | L318V | high |
| TCGA-A5-A0G2-01 | **UCEC** | N277I, C760Y, A427T, S865F | high |
| TCGA-A5-A0VP-01 | **UCEC** | P565L | high |
| TCGA-A5-A2K3-01 | **UCEC** | V502A, R267Q | high |
| TCGA-A5-A2K5-01 | **UCEC** | N836D | low |
| TCGA-A6-6654-01 | **COAD** | K743R | low |
| TCGA-A8-A09Z-01 | **BRCA** | I677T | low |
| TCGA-AA-3977-01 | **COAD** | R476I, K467N | high |
| TCGA-AA-A00N-01 | **COAD** | R563Q, A886T | high |
| TCGA-AA-A010-01 | **COAD** | S766Y, R926Q | high |
| TCGA-AC-A23H-01 | **BRCA** | S338C | high |
| TCGA-AG-A002-01 | **READ** | E213K, M839L, K499Q | high |
| TCGA-AJ-A3EL-01 | **UCEC** | L980Rfs*8 | low |
| TCGA-AN-A0XW-01 | **BRCA** | S500* | high |
| TCGA-AP-A051-01 | **UCEC** | A357T, M839I | low |
| TCGA-AP-A056-01 | **UCEC** | N96H, D803Y, R926Q | high |
| TCGA-AP-A059-01 | **UCEC** | R426S | low |
| TCGA-AP-A0LF-01 | **UCEC** | S832N | high |
| TCGA-AP-A0LP-01 | **UCEC** | W264R | high |
| TCGA-AP-A1DV-01 | **UCEC** | D672Y | high |
| TCGA-AX-A05Z-01 | **UCEC** | K649T | high |
| TCGA-AX-A06F-01 | **UCEC** | K999T | high |
| TCGA-AX-A0J1-01 | **UCEC** | A837V | high |
| TCGA-AX-A1CE-01 | **UCEC** | K458N, D269N | high |
| TCGA-AX-A2HJ-01 | **UCEC** | S279Lfs*14 | low |
| TCGA-AZ-4315-01 | **COAD** | E401D, K742T | low |
| TCGA-B5-A0JY-01 | **UCEC** | R563Q, E38*, L809V | high |
| TCGA-B5-A11E-01 | **UCEC** | N935T | high |
| TCGA-B5-A11N-01 | **UCEC** | M339I | low |
| TCGA-B5-A1MR-01 | **UCEC** | E517*, R801I | high |
| TCGA-B5-A1MX-01 | **UCEC** | V108A | high |
| TCGA-B5-A3FC-01 | **UCEC** | D56N | low |
| TCGA-B6-A0RU-01 | **BRCA** | Q86del | low |
| TCGA-BA-4076-01 | **HNSC** | L171F, K413Q | high |
| TCGA-BK-A6W3-01 | **UCEC** | I130L | low |
| TCGA-BR-4184-01 | **STAD** | R384H | low |
| TCGA-BR-7851-01 | **STAD** | R661H | high |
| TCGA-BR-8060-01 | **STAD** | A687G | high |
| TCGA-BR-8078-01 | **STAD** | R267* | high |
| TCGA-BR-8361-01 | **STAD** | H228Qfs*2 | low |
| TCGA-BR-8382-01 | **STAD** | V58I | low |
| TCGA-BR-8680-01 | **STAD** | T238P | high |
| TCGA-BS-A0UF-01 | **UCEC** | K95N, K823T | high |
| TCGA-BS-A0UV-01 | **UCEC** | K325N | low |
| TCGA-CA-6717-01 | **COAD** | E259* | high |
| TCGA-CR-7365-01 | **HNSC** | I369M | low |
| TCGA-CV-7568-01 | **HNSC** | E321K | low |
| TCGA-D1-A103-01 | **UCEC** | V10I | low |
| TCGA-D1-A17M-01 | **UCEC** | X356_splice | low |
| TCGA-D3-A8GM-06 | **SKCM** | P35L | low |
| TCGA-D5-6928-01 | **COAD** | R620C | high |
| TCGA-D8-A1XQ-01 | **BRCA** | X298_splice | low |
| TCGA-D9-A148-06 | **SKCM** | G652* | high |
| TCGA-D9-A149-06 | **SKCM** | L699M | low |
| TCGA-DA-A1IB-06 | **SKCM** | K847N | low |
| TCGA-DF-A2KN-01 | **UCEC** | I1007T, P239Q | low |
| TCGA-DF-A2KU-01 | **UCEC** | V307L, Q204H | low |
| TCGA-DI-A1BU-01 | **UCEC** | L1009S | high |
| TCGA-DK-A2I2-01 | **BLCA** | G542V | high |
| TCGA-DK-A2I4-01 | **BLCA** | I481M | high |
| TCGA-DQ-5624-01 | **HNSC** | D513H | high |
| TCGA-DU-6392-01 | **LGG** | E822*, E213*, A357T, V778I, T906I | high |
| TCGA-DU-A76L-01 | **LGG** | L909R | high |
| TCGA-DX-A48R-01 | **SARC** | A141T | low |
| TCGA-DY-A1H8-01 | **READ** | E155* | high |
| TCGA-E6-A1LX-01 | **UCEC** | E612*, V787A, K888N | high |
| TCGA-EB-A24D-01 | **SKCM** | X872_splice | low |
| TCGA-EE-A2GE-06 | **SKCM** | G696V | high |
| TCGA-EE-A2GI-06 | **SKCM** | G696W | high |
| TCGA-EE-A2M8-06 | **SKCM** | P731L | low |
| TCGA-EE-A2MU-06 | **SKCM** | S161L | low |
| TCGA-EE-A3AC-06 | **SKCM** | G152E | high |
| TCGA-EE-A3J7-06 | **SKCM** | R157K | low |
| TCGA-EI-6917-01 | **READ** | S397N | low |
| TCGA-EK-A3GK-01 | **CESC** | S893* | high |
| TCGA-EO-A22R-01 | **UCEC** | D215G | high |
| TCGA-EO-A22S-01 | **UCEC** | R819C | low |
| TCGA-EO-A22T-01 | **UCEC** | R514H | high |
| TCGA-EO-A22U-01 | **UCEC** | R384H | low |
| TCGA-EO-A22X-01 | **UCEC** | E213*, K6N | high |
| TCGA-EO-A3AV-01 | **UCEC** | K649T | high |
| TCGA-EO-A3B0-01 | **UCEC** | E259*, R741I | high |
| TCGA-F4-6460-01 | **COAD** | D94G | high |
| TCGA-F5-6814-01 | **READ** | K888N, D56Y | high |
| TCGA-FI-A2D5-01 | **UCEC** | R580I, V962A | high |
| TCGA-FS-A1ZG-06 | **SKCM** | R713L | high |
| TCGA-FS-A1ZM-06 | **SKCM** | W11L | low |
| TCGA-FW-A3R5-06 | **SKCM** | P135S | high |
| TCGA-GN-A266-06 | **SKCM** | S884F | high |
| TCGA-GN-A26A-06 | **SKCM** | L248I | high |
| TCGA-JW-A5VL-01 | **CESC** | S400* | high |
| TCGA-KK-A7AY-01 | **PRAD** | N102S | high |
| TCGA-KQ-A41R-01 | **BLCA** | Q792K | low |
| TCGA-L5-A8NQ-01 | **ESCA** | E738Q | low |
| TCGA-MP-A4SV-01 | **LUAD** | S833L | high |
| TCGA-ND-A4WC-01 | **UCS** | F506V, K888N | high |
| TCGA-OR-A5J8-01 | **ACC** | I665F | high |
| TCGA-OR-A5LR-01 | **ACC** | I689T | high |
| TCGA-PG-A6IB-01 | **UCEC** | T50S | low |
| TCGA-PL-A8LV-01 | **BRCA** | A460S | low |
| TCGA-T3-A92M-01 | **HNSC** | S874C | high |
| TCGA-UC-A7PG-01 | **CESC** | I1003M | high |
| TCGA-VQ-A8P2-01 | **STAD** | X330_splice | low |
| TCGA-VS-A9U5-01 | **CESC** | Q496* | high |
| TCGA-VS-A9UZ-01 | **CESC** | S485F | high |
| TCGA-W3-AA1V-06 | **SKCM** | R644K | high |
| TCGA-XF-AAMX-01 | **BLCA** | S447F | low |
| TCGA-XK-AAIW-01 | **PRAD** | A443G | low |
| TCGA-Z2-A8RT-06 | **SKCM** | S378L | low |
| TCGA-ZF-A9RC-01 | **BLCA** | E806K | low |
| TCGA-ZJ-A8QR-01 | **CESC** | P814L | low |

**Supplementary Table S11.** Somatic mutations, single-nucleotide variants (SNVs) and short insertions/deletions (indels), identified on *HLTF* across TCGA 143 samples and retrieved from cBioPortal. For each sample, the corresponding cancer type, predicted functional impact (Impact), and protein-level amino acid change (Protein change) are reported.

| **Cancer type** | **Promoter-proximal** | **HLTF expression** | **Impact on prognosis** |
| --- | --- | --- | --- |
| **COAD** | Increased methylation | Decreased | Not significant |
| **READ** | Increased methylation | Decreased | Not significant |
| **STAD** | Increased methylation | Decreased | Not significant |
| **HNSC** | Increased methylation | Decreased | Not significant |
| **ESCA** | Increased methylation | Decreased | Not significant |
| **DLBCL** | Increased methylation | Not significant | Not significant |

**Supplementary Table S12.** Summary of the correlation between the methylation on *HLTF* promoter-proximal region with its mRNA expression and survival. For all cancer types showing a statistically significant over-representation of patients with increased HLTF promoter methylation, the corresponding effects on HLTF expression and prognosis are reported. “Not significant” indicates that no statistically significant impact was detected.

| **Cancer type** | **Promoter-distal** | **HLTF expression** | **Impact on prognosis** |
| --- | --- | --- | --- |
| **CESC** | Increased methylation | Increased (cg02059813 only) | Not significant |
| **TGCT** | Increased methylation | Not significant | Not significant |
| **LUAD** | Increased methylation | Increased (cg02059813 only) | Not significant |
| **STAD** | Increased methylation | Not significant | Not significant |
| **SARC** | Increased methylation | Decreased (cg21089667 only) | Not significant |

**Supplementary Table S13.** Summary of the correlation between the methylation on *HLTF* promoter-distal region with its mRNA expression and survival. For all cancer types showing a statistically significant over-representation of patients with increased HLTF promoter methylation, the corresponding effects on HLTF expression and prognosis are reported. “Not significant” indicates that no statistically significant impact was detected.

| **Cancer type** | **CNA class** | **Impact on HLTF expression** | **Impact on prognosis** |
| --- | --- | --- | --- |
| **AML** | 3 | Not-changed | Not significant |
| **ACC** | 4 | Not-changed- | Not significant |
| **BLCA** | 1 | Increased (FC 0.34, FDR 6.70e-11) | Not significant |
| **LGG** | 3 | Not-changed- | Not significant |
| **BRCA** | 4 | Not-changed- | Not significant |
| **CESC** | 1 | Increased (FC 0.18, FDR 0.006) | Not significant |
| **CHOL** | 4 | Not-changed- | Not significant |
| **COAD** | 4 | Not-changed- | Not significant |
| **DLBCL** | 4 | Not-changed- | Not significant |
| **ESCA** | 1 | Not-changed- | Not significant |
| **GBM** | 4 | Not-changed- | Not significant |
| **HNSC** | 1 | Increased (FC 0.63, FDR 5.98E-12) | Not significant |
| **KICH** | 4 | Not-changed- | Not significant |
| **KIRC** | 4 | Not-changed- | Not significant |
| **KIRP** | 4 | Not-changed- | Not significant |
| **LIHC** | 4 | Not-changed- | Not significant |
| **LUAD** | 4 | Not-changed- | Not significant |
| **LUSC** | 1 | Increased (FC 0.25, FDR 6.53e-07) | Not significant |
| **MESO** | 4 | Not-changed- | Not significant |
| **OV** | 1 | Increased (FC 0.27, FDR 3.16e-08) | Not significant |
| **PAAD** | 4 | Not-changed- | Not significant |
| **PCPG** | 2 | Decreased (FC -0.37, FDR 3.35e-08) | Not significant |
| **PRAD** | 4 | Not-changed- | Not significant |
| **READ** | 4 | Not-changed- | Not significant |
| **SARC** | 4 | Not-changed- | Not significant |
| **SKCM** | 4 | Not-changed- | Not significant |
| **STAD** | 4 | Not-changed- | Not significant |
| **TGCT** | 4 | Not-changed- | Not significant |
| **THYM** | 3 | Not-changed- | Not significant |
| **THCA** | 3 | Not-changed- | Not significant |
| **UCS** | 1 | Increased (FC 0.33, FDR 0.008) | Not significant |
| **UCEC** | 4 | Not-changed- | Not significant |
| **UVM** | 2 | Not significant | Decreased survival |

**Supplementary Table S14.** Impact of each *HLTF* CNA category as defined in **Figure 2A**, (1) Gain/Amplification, (2) Heterozygous loss, (3) Low CNA frequency, and (4) Balanced gain/loss, on gene expression and prognosis. Numbers in parenthesis indicate Log_2_(FC) and FDR values [Massey 1951: doi.org/10.1080/01621459.1951.10500769].

**
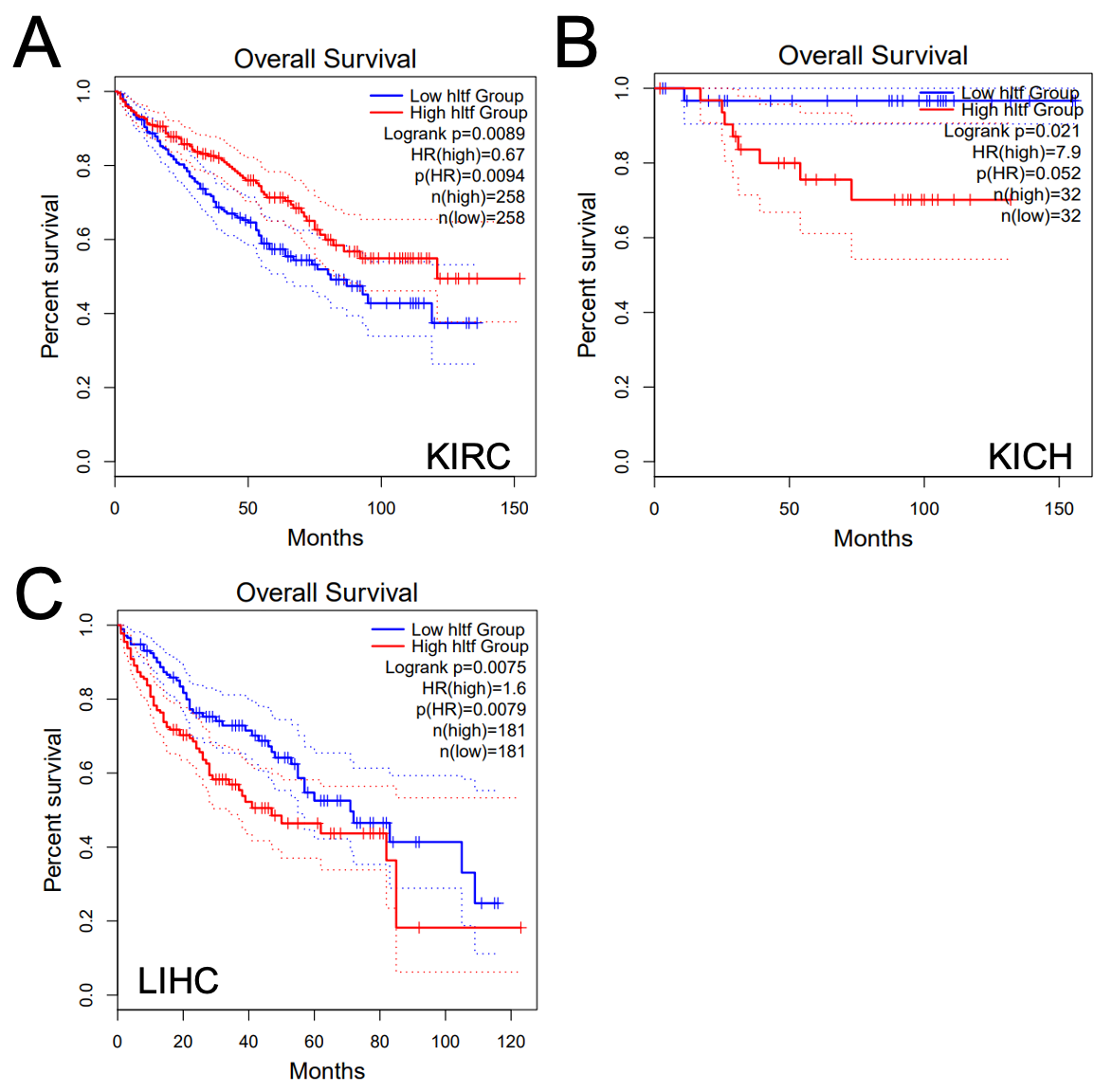
**

**Supplementary Figure S1. A.** Kaplan-Meier plot of overall survival (OS) extracted from KIRC tumour patients either with high (red) or low (blue) *HLTF* mRNA expression. Sample numbers and statistics are indicated. **B.** Same plot as in panel A for KICH. **C.** Same plot as in panel A for LIHC.


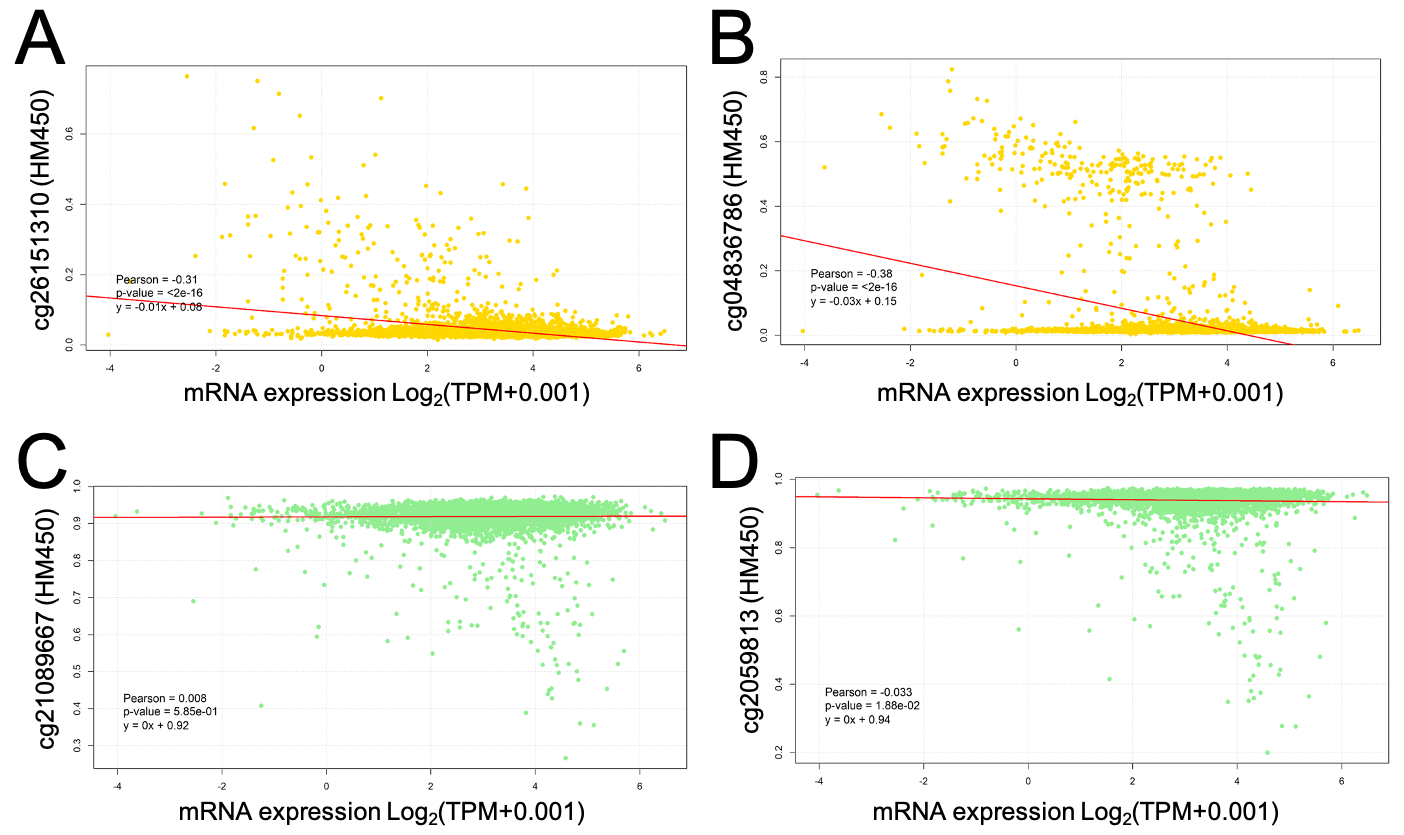


**Supplementary Figure S2.** Correlation between *HLTF* methylation (β values) and its mRNA (Log_2_(TPM+0.001)) along with the relative metrics (Pearson, p-value and slope) across the Illumina HumanMethylation450 arrays: the two promoter-proximal cg26151310 (**A**) and cg04836786 (**B**) in yellow, and the two promoter-distal cg21089667 (**C**) and cg02059813 (**D**) in green.


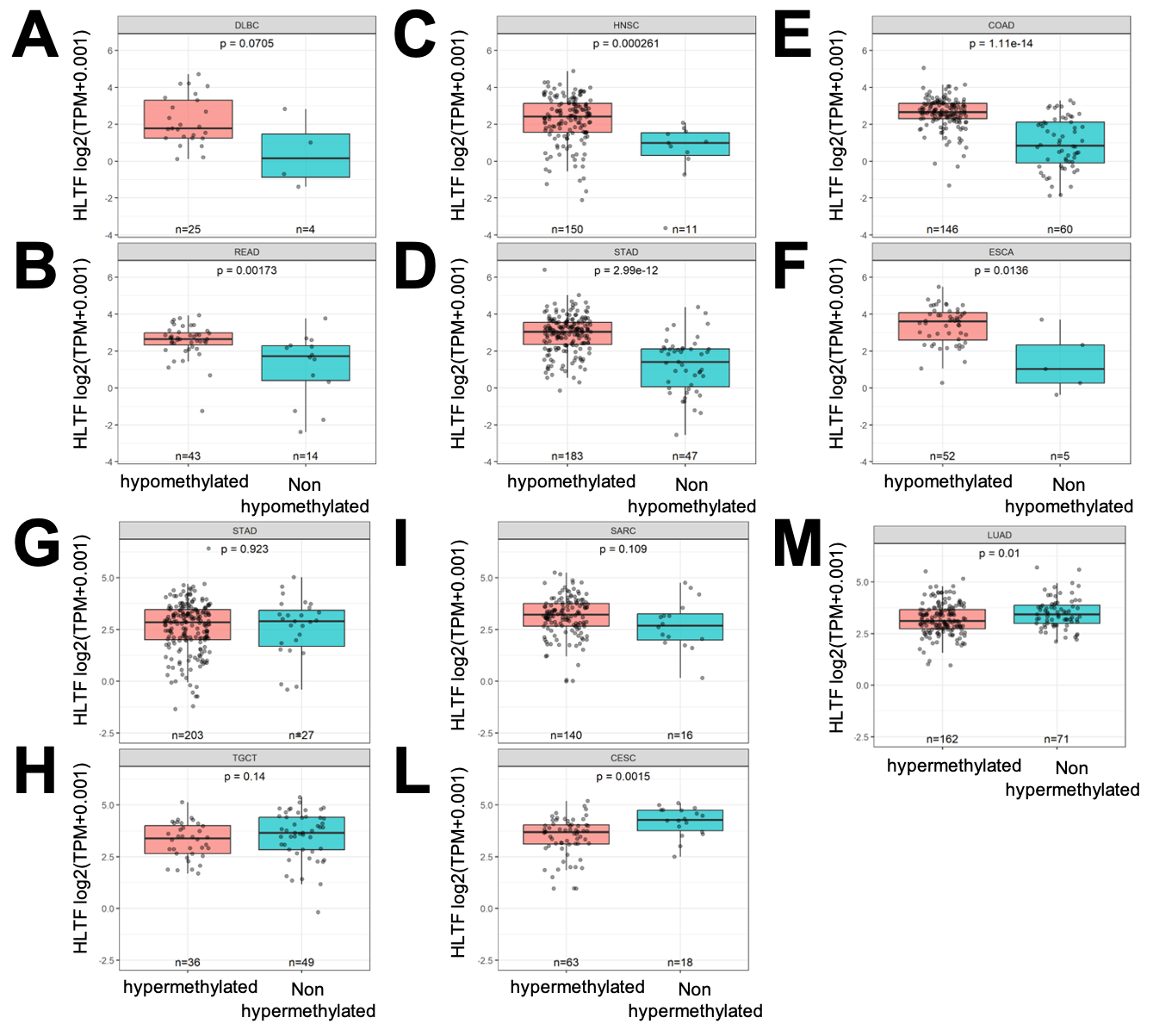


**Supplementary Figure S3.** Association between *HLTF* promoter methylation (β values) and its mRNA expression across each cancer type. Those cancers displaying non-hypomethylated (red) versus hypomethylated samples (blue) mapped at the promoter-proximal probes are **A.** DLBCL, **B.** READ, **C** HNSC, **D.** STAD, **E.** COAD, **F.** ESCA. Those cancers displaying hypermethylated (red) versus non-hypermethylated samples (blue) mapped at the promoter-distal probes are **G.** STAD, **H.** TGCT, **I.** SARC, **L.** CESC, and **M.** LUAD. For each condition, either hypo-/non-hypo- or hyper-/non-hyper-, the number of samples considered is indicated.


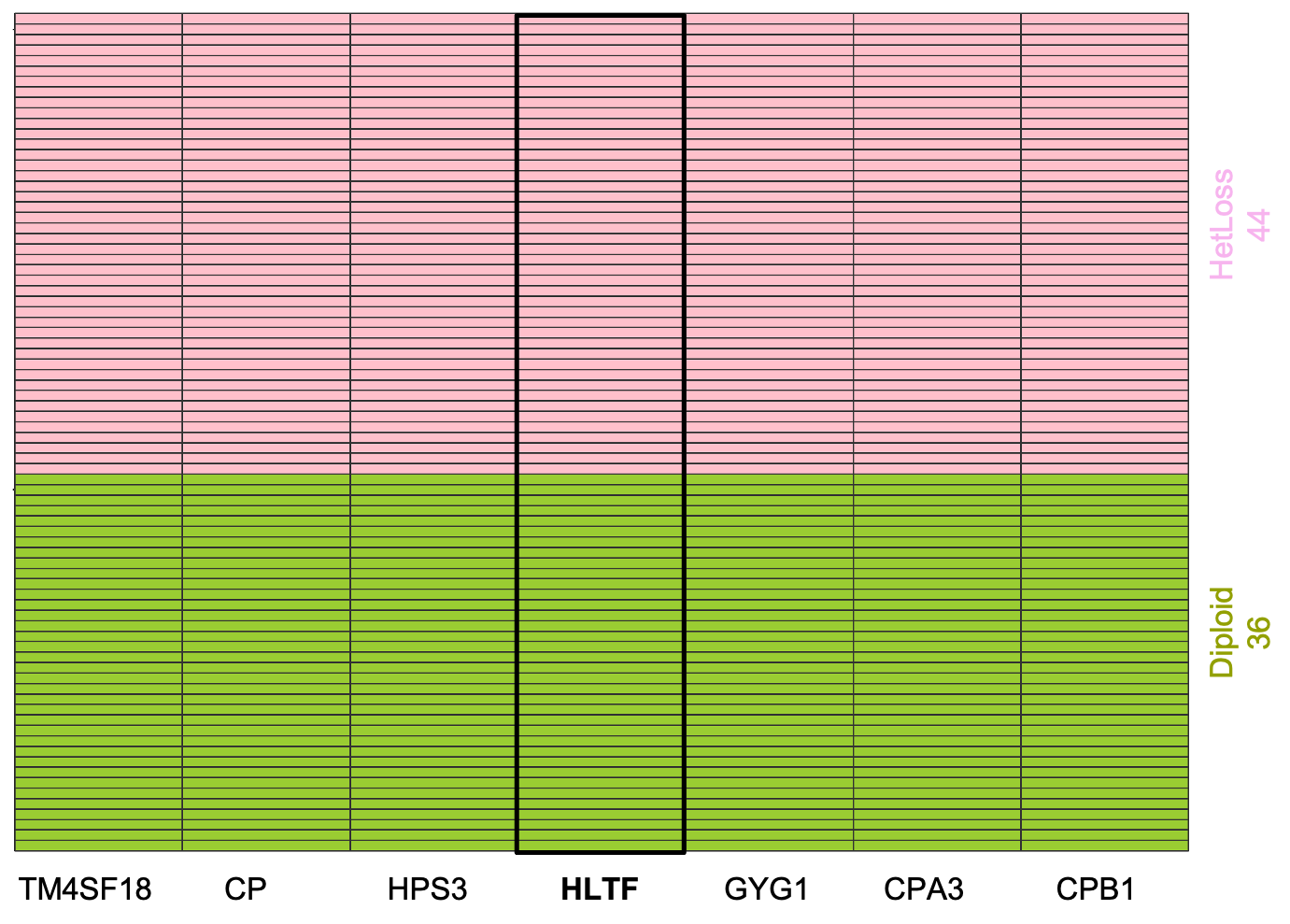


**Supplementary Figure S4.** Heatmap representing the distribution of samples (numbers indicated) obtained from UVM and containing either *HLTF* (squared box) CNA HetLoss (light pink) or diploid (forest green). The analysis is extended to the neighbouring downstream genes, namely HPS3, CP, and TM4SF18, and upstream genes, namely GYG1, CPA3, and CPB1 on chromosome 3. The RefSeq annotation 10.4 genomic coordinates (GRCh37/hg19 assembly) were converted to GRCh38/hg38 assembly using the UCSC LiftOver tool [Perex et al 2025: doi: 10.1093/nar/gkae974].

**
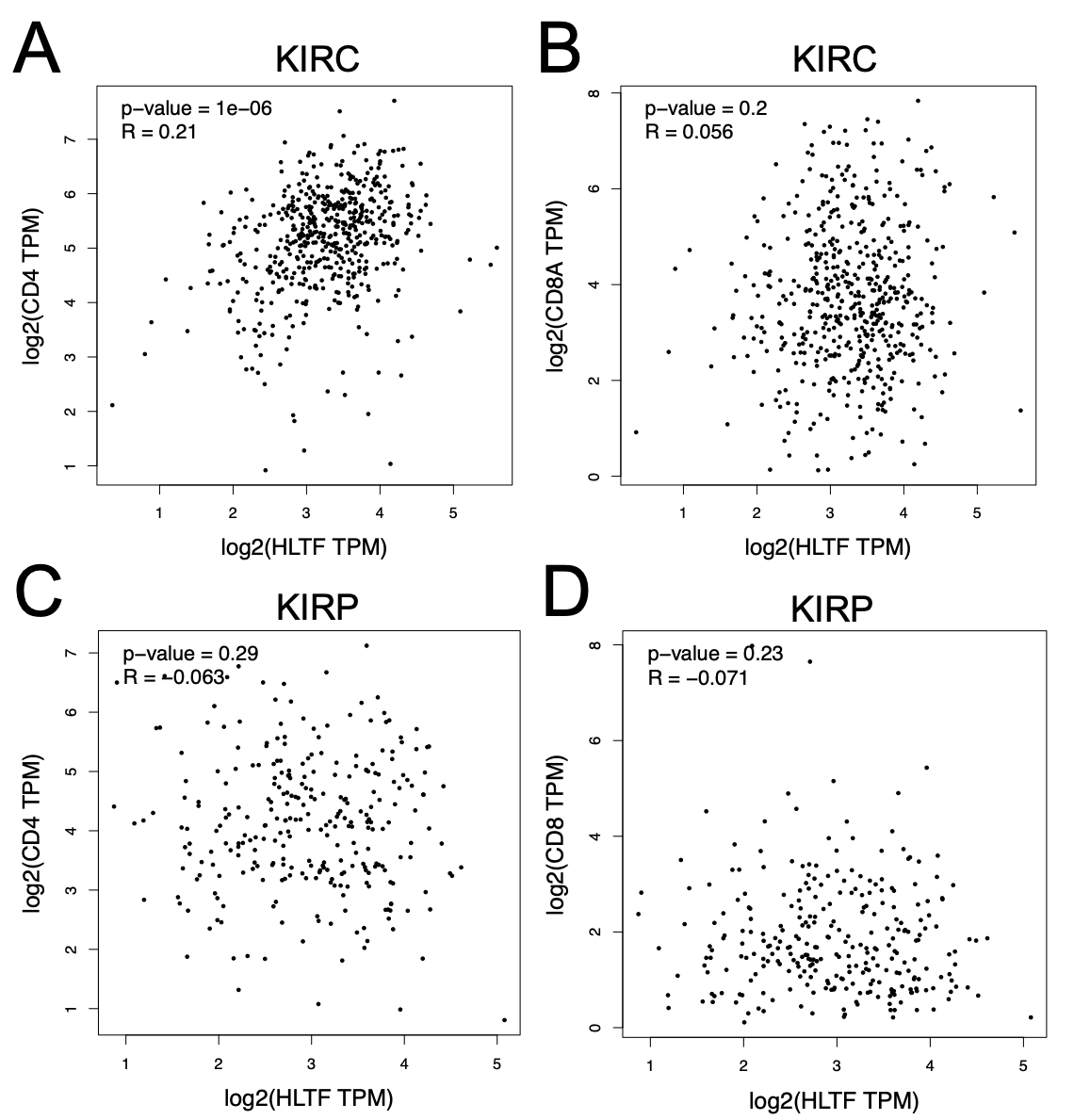
**

**Supplementary Figure S5. A.** mRNA coexpression patterns in transcript per million (TPM) and in KIRC of HLTF versus CD4. R: Pearson correlation coefficient; p-value. **B.** Same pattern as in panel A of HLTF versus CD8. **C.** Same pattern as in panel A in KIRP. **D.** Same pattern as in panel B in KIRP.
